# Deep Learning Prediction of Metastasis in Locally Advanced Colon Cancer Using Binary Histologic Tumor Images

**DOI:** 10.3390/cancers13092074

**Published:** 2021-04-25

**Authors:** Stefan Schiele, Tim Tobias Arndt, Benedikt Martin, Silvia Miller, Svenja Bauer, Bettina Monika Banner, Eva-Maria Brendel, Gerhard Schenkirsch, Matthias Anthuber, Ralf Huss, Bruno Märkl, Gernot Müller

**Affiliations:** 1 Institute of Mathematics, Augsburg University, 86159 Augsburg, Germany; tobias.arndt@uka-science.de (T.T.A.); gernot.mueller@math.uni-augsburg.de (G.M.); 2 General Pathology and Molecular Diagnostics, Medical Faculty, 86156 Augsburg, Germany; benedikt.martin@uk-augsburg.de (B.M.); Silvia.Miller@uk-augsburg.de (S.M.); svenja.bauer@uk-augsburg.de (S.B.); bettina-monika@gmx.de (B.M.B.); Eva-Maria.Brendel@uk-augsburg.de (E.-M.B.); Ralf.Huss@uk-augsburg.de (R.H.); bruno.maerkl@uka-science.de (B.M.); 3 Tumor Data Management, University Hospital of Augsburg, 86156 Augsburg, Germany; Gerhard.Schenkirsch@uk-augsburg.de; 4 General, Visceral, and Transplantation Surgery, University Hospital of Augsburg, 86156 Augsburg, Germany; Matthias.Anthuber@uk-augsburg.de

**Keywords:** colon cancer, tumor stroma ratio, pattern, deep learning, tumor architecture, prognostic biomarker

## Abstract

**Simple Summary:**

Deep learning methods are increasingly being applied for tissue classification to improve diagnosis and optimize therapy stratification. In this study, we developed the Binary ImaGe Colon Metastasis classifier (BIg-CoMet), a semi-guided approach for the stratification of colon cancer patients into two risk groups according to the occurrence of distant metastasis, using an InceptionResNetV2-based deep learning model trained on binary images. For a validation collective (*n* = 128), we showed that BIg-CoMet was able to stratify patients appropriately. The predicted high-risk group showed a worse clinical course for being metastasis-free, and the risk group was a prognostic factor for the occurrence of metastasis. These results were also found for both Union Internationale Contre le Cancer (UICC) subgroups. We demonstrated that Big-CoMet is useful for the stratification of colon cancer patients into risk groups based on images reflecting tumor architecture.

**Abstract:**

In this study, we developed the Binary ImaGe Colon Metastasis classifier (BIg-CoMet), a semi-guided approach for the stratification of colon cancer patients into two risk groups for the occurrence of distant metastasis, using an InceptionResNetV2-based deep learning model trained on binary images. We enrolled 291 colon cancer patients with pT3 and pT4 adenocarcinomas and converted one cytokeratin-stained representative tumor section per case into a binary image. Image augmentation and dropout layers were incorporated to avoid overfitting. In a validation collective (*n* = 128), BIg-CoMet was able to discriminate well between patients with and without metastasis (AUC: 0.842, 95% CI: 0.774–0.911). Further, the Kaplan–Meier curves of the metastasis-free survival showed a highly significant worse clinical course for the high-risk group (log-rank test: *p* < 0.001), and we demonstrated superiority over other established risk factors. A multivariable Cox regression analysis adjusted for confounders supported the use of risk groups as a prognostic factor for the occurrence of metastasis (hazard ratio (HR): 5.4, 95% CI: 2.5–11.7, *p* < 0.001). BIg-CoMet achieved good performance for both UICC subgroups, especially for UICC III (*n* = 53), with a positive predictive value of 80%. Our study demonstrates the ability to stratify colon cancer patients via a semi-guided process on images that primarily reflect tumor architecture.

## 1. Introduction

Colon cancer is among the three most prevalent cancer types in Germany. According to the Robert-Koch Institute, in 2016 colon cancer was diagnosed in every eighth cancer patient [1]. Further, around 60,000 incidents and about 25,000 deaths were recorded, underlining the high mortality rate of this cancer type [1]. Hence, a correct staging of cancer tissue is essential for the right choice of therapy and for the estimation of the survival chance of the patient.

The classification of colon cancer relies mainly on the Union Internationale Contre le Cancer (UICC) staging, which serves as the leading tool for therapy stratification and prognostic estimations. This system is based on the local extent of the tumor and the occurrence or absence of regional and distant metastases [2]. Although fully accepted in daily practice, it does not correlate perfectly with the clinical course of the individual cases. Stage II cases are known to have an excellent prognosis. Adjuvant therapy has been shown to be of no benefit to those patients and is offered only in cases with additional risk factors [3]. However, about 20% of these cases show adverse behavior [4]. The effectiveness of adjuvant chemotherapy in Stage III colorectal cancer has been proven, resulting in a relative survival benefit of about 30%. However, some patients do not benefit from this therapy but suffer from side effects [5,6]. This underlines the urgent need for affordable, reliable biomarkers that are ideally easily integrated into the routine diagnostics. Many promising biomarkers, such as infiltration typing, microsatellite status, tumor-infiltrating immune cells, and poorly differentiated clusters, have been suggested [7,8,9,10]. Of these, only tumor budding has been recently implemented in routine diagnostics. It has been generally recommended in node-negative cases since 2016 after the publication of the results of a consensus meeting in Bern [11]. Further, the determination of the tumor to stroma ratio (TSR) is receiving increasing attention in the search for further biomarkers [12,13,14]. TSR is the ratio between the area of invasive neoplastic cells and the surrounding nonneoplastic tissue consisting of mesenchymal, myofibroblastic, and immune cells. Usually, the TSR is evaluated on the basis of hematoxylin–eosin (H&E) stained slides by estimation [14]. A threshold of 50% is generally accepted, and stroma-rich tumors have been shown as prognostically unfavorable. We and others have applied advanced methods to increase the precision of this task [15,16,17]. Based on pan-cytokeratin staining, we produced binary images that allowed an exact quantification of the tumor stroma ratios in colon cancers [17]. Next to the simple area measurement of tumors and stroma, we developed the hypothesis that very heterogeneous architectures of the tumor glands represent the phenotypes of tumors with different biological behaviors. Pattern recognition is one of the main skills of pathologists. However, methods of artificial intelligence are also perfectly suited for such image-based stratifications and have the potential to identify new prognostically meaningful patterns that have so far escaped the eye of the pathologists. Therefore, independent algorithms can surpass conventional methods in terms of their prognostic significance.

In recent years, convolutional neural networks (CNN) and other deep learning algorithms have been established as state-of-the-art methods for a wide field of image classification tasks. CNNs for the quantification of tumor stroma proportion have already been developed [18], and it has been shown that tumor budding can be validly determined using machine learning methods [19]. For a comprehensive review of deep learning in colon cancer, we refer to Pacal et al. [20]. However, the number of studies that have examined a direct prediction of the outcome based on histological tumor images using deep learning is very limited, and these are mainly based on H&E images.

The aim of the present study was to investigate the prognostic value (primary endpoint: occurrence of distant metastasis) of deep learning in locally advanced colon cancer based on histologic tumor images using a semi-guided approach. We intentionally reduced the pathologist’s input into the deep learning analysis to yield a semi-guided approach that allowed the model to focus on certain features in the data (e.g., the shape of tumor border). In this study, we implemented this approach using pure black and white histologic images based on pan-cytokeratin staining of a specific tumor region as input information instead of images of whole H&E slides of the tumor. It seems obvious that the proportion of tumor stroma can be easily recognized here, as can the architecture of the tumor by the human eye. This approach using black and white images is, among others, blinded to more sophisticated morphological features such as nuclear atypia or mitosis, as well as the composition of the inflammatory reaction. Therefore, the aim of this study was to evaluate whether a CNN based on substantially reduced image-based information can stratify locally advanced colon cancer into prognostically different groups.

Our main hypothesis was that the occurrence of distant metastasis can be predicted using deep learning with higher significance than with the established criteria despite reduced tumor image information.

## 2. Materials and Methods

This study conforms to the REMARK guidelines [21].

### 2.1. Case Collectives

Retrospectively, we investigated two independent case collectives of locally advanced pT3/4, N±, M0, and R0 colon adenocarcinomas not otherwise specified (special types, such as mucinous carcinoma, medullary carcinoma, and signet cell carcinoma, were excluded) treated in a single center (University Hospital Augsburg). Inclusion criteria for the training cohort (*n* = 163) were operative treatment between 2012 and 2016 and the occurrence of distant metastases or documented metastasis-free survival of at least five years. The validation set included 128 patients who underwent surgery between January 2002 and December 2011, fulfilling the same inclusion criteria as the training cohort. Follow-up data for all cases were provided by the Tumor Data Management of the University Hospital Augsburg, complemented with data of the patient files. The patients were treated in accordance with the valid guidelines at that time.

### 2.2. Sample Preparation and Immunohistochemistry

After surgery, the patient’s tumor tissue was fixed immediately in 4% buffered formalin for at least 12 h and then embedded in paraffin. The slides investigated were made of 3 µm FFPE tissue block sections. The diagnoses were retrieved from the pathological reports. Tumor budding classification was performed according to the criteria defined by the International Tumor Budding Consensus Conference (ITBCC) [11], as previously described [22]. Right-sided tumors were defined up to the left colonic flexure (excluded). The tumor proportion and tumor proportion group classifications were defined as recently published [17].

### 2.3. Sample Preparation and Digital Processing

**Figure 1:**
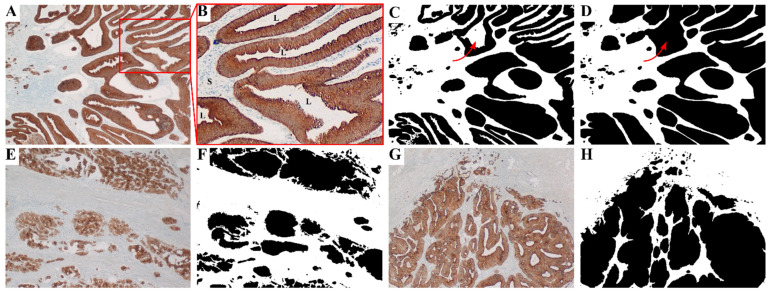
The image preparation process is illustrated in (**A**–**D**), starting with a stained image (**A**); then the intermediary ImageJ output (**C**); and the final image, which was manually corrected for lumen not filled by the algorithm (**D**). One such lumen is indicated with an arrow for better visibility. In (**B**), an enlarged section of (**A**) with enhanced contrast is shown to illustrate the difference between lumen, denoted by L, and stroma, denoted by S. Note that the cell nuclei in the stroma were stained blue, whereas the lumen remained white. Further shown are images of patients without metastasis (**E**), with metastasis (**G**), and the respective binary images fed to the network (**F**,**H**). The predicted risk of distant metastasis by BIgCoMet was 9.6% for F (low-risk group) and 85.5% for H (high-risk group).

Detailed processing has been described before [17]. In brief, the whole H&E slide was viewed, and the best fitting region at the point of deepest infiltration that contained no artifacts of blood vessels, necrosis, or other special type was selected. In the next step, a rectangular region with a field size of 3.58 mm^2^ was extracted from the whole slide, which contained tumor cells at all borders of the image field. The selection process for each region was performed using a microscope (Olympus, BX43F, Tokyo, Japan) with an attached camera with a connection to a computer (ProgRes Speed XTcore5 with combined software: Capture Pro 2.9.0.1) [17]. All images were immunohistochemically stained against cytokeratin (cytokeratin AE1/AE3) to highlight tumor tissue. We prepared the anti-cytokeratin AE1/AE3 immunostaining according to our routine protocol (immunostainer, Roche Benchmark Ultra; DAB Opti View IHC Detection Kit antibody, cell marque™, Mannheim, Germany, monoclonal mouse antibody; dilution 1:500). If there was already an anti-cytokeratin-stained slide available from the previous routine diagnostics, we used this instead of performing a new immunostaining. Tissues containing the tumor reacted immunohistochemically and were marked brown. In further steps, the obtained image was processed with the open-source image software ImageJ (Version 1.48 v) [23,24]. The differentiation between stained tumors and stroma was accomplished via automated thresholding, which was independent of manually chosen hyperparameters. For this step, we used the command “run (“make binary”)*”* in ImageJ, resulting in black and white images. A sensibility analysis proved that the automatically selected threshold was well suited to translating images into black and white. When deviations of the optimal threshold were used, we obtained a similar tumor architecture, but noise was introduced in the images because parts of the tumor remained unrecognized or stroma was incorrectly classified as tumor (Figure S1). After translation of the image into binary values, the lumen of the tumor cells was filled automatically (command: run (“fill holes”)). This is important because otherwise the holes would be assigned as stromal parts. If lumen had not been filled correctly by the software algorithm, the resulting image was manually improved by filling the remaining gaps using a drawing tool. Tumor lumens are characterized in particular by the lack of cell nuclei that appear slightly blue, and they are completely enclosed by dark tumor cells. In the edge areas of the image section, they can appear pseudo-open because a part of the enclosing cells is not covered by the image. A detailed description of this process is given in [17], and examples are shown in Figure 1. The produced image was used as an input for our machine learning model.

### 2.4. Deep Learning Architecture

The neural network described in the following section was implemented in Python 3.6.9 using the Keras framework supplied by the TensorFlow 2.3.1 platform and trained using a Nvidia Tesla V100 GPU.

### 2.5. Feature Extraction

The binary images were downscaled to 680 × 840 pixels by a factor of three to improve the learning performance of the algorithm, and the pixel values were normalized to values between 0 and 1 from 0 and 255. The images of the training and testing sets were split (80/20%). These images were then further reduced in size by a factor of three using a convolution layer of three 20 × 20 filters with a stride of three and padding, resulting in a 216 × 287-pixel image with three channels. On the output, a hyperbolic tangent activation function is applied. We used the InceptionResNetV2 network [25], pretrained on images from the ImageNet challenge, to extract features from the downscaled images in the form of 1536 5 × 7 feature maps. The output was pooled using GlobalAveragePooling with a stride of two.

This was followed by a classifier consisting of two fully connected layers with Relu activation functions, containing 256 nodes in the first layer and 64 nodes in the second layer, and a fully connected output layer containing two nodes with a SoftMax activation function. The final output predicted the probabilities for metastasis or no metastasis.

### 2.6. Avoiding Overfitting

As the training set was of a rather small size, the model needed to be prevented from overfitting the data. To achieve this, several measures were employed. Keras supplies a toolbox for altering input images (ImageDataGenerator), which allows geometric manipulation of the input images [26]. As the rotation and position of the cancer in the image, as well as mirroring, should have no effect on the label, we used the implementation of such random augmentations to generate altered images for each training epoch.

The values for these augmentations were uniformly drawn from ±15 degrees for the rotation angle, ±10% for the width and height shift, and 0–1 degrees for the shear. The voids created in the image by these augmentations were filled using reflections of the image at its borders.

**Figure 2:**
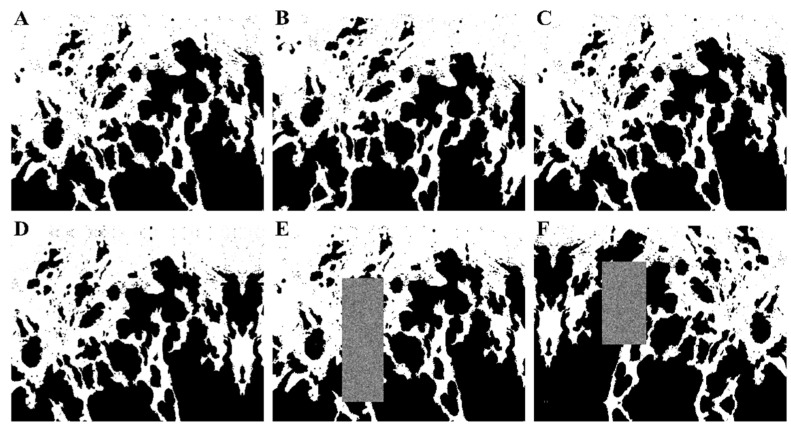
Different augmentations of the original image (**A**) through rotation (**B**), shearing (**C**), shifting (**D**), random erasing (**E**), and a combination of all methods (**F**).

Random rectangular sections in the image were replaced by noise [27]. For this step, the Python implementation by Yusuke Uchida and Kosuke Takeuchi was used [28]. This further prevented the network from “memorizing” the data. The noise was generated using uniformly distributed variables for pixel values, which were then smoothed using a Gaussian blur with a sigma of 1. Examples of the augmentations of the images are given in Figure 2.

To emulate ensemble learning and further reduce the variance of the model, dropout [29] was applied to the output of the InceptionResNetV2 as well as the layers in the classifier (except output). The values chosen for dropout are 10% for the output of the InceptionResNetV2, 20% for the first fully connected layer, and 10% for the second one.

### 2.7. Training

Only the initial scaling convolution layer and the classifier were trained on the data described above, while the weights of InceptionResNetV2 were fixed to the pretrained values. Training was carried out using categorical cross entropy as a loss function and RMSprop with a learning rate of 0.0005 as optimizer. The network was trained for 300 epochs using batches of 21 and validated after each epoch on the test data. After each epoch, the network’s performance was evaluated using the test data. The model with the best cross entropy loss was chosen for further analysis.

### 2.8. Definition of BIg-CoMet

For every patient in the validation set, the probability for occurrence of metastasis was predicted by the neural network and assigned as the score of the patient, ranging from 0 to 1. The patients were divided into a low- and a high-risk group by a cutoff of 0.5, which was reasoned by the cutoff of the model during training and found to be meaningful for the training dataset. We named the binary risk classifier based on the deep learning approach with the preprocessing procedure Binary ImaGe Colon Metastasis classifier (BIg-CoMet).

### 2.9. Statistics

Clinicopathological data were described by counts and percentages for categorized parameters and mean and standard deviation for continuous parameter. For each image in the independent test set, the predicted probability of developing metastasis was computed separately. Age, sex, and clinicopathological variables were compared between groups with low and high-risk with a *t*-test or a Wilcoxon-Mann-Whitney test for continuous variables and a Chi-square test or an exact Fisher test for categorical variables.

For each risk group, we computed Kaplan–Meier curves for metastasis-free survival and compared the groups using a log-rank test to identify the algorithms’ ability to stratify patients with respect to the rise of metastasis. Further, we fitted univariate Cox proportional hazard regression models for the metastasis-free survival of the test set with the risk group and other clinicopathological variables as independent variables to select potentially independent risk factors for a multivariable model.

A multivariable Cox regression model for metastasis-free survival was used to identify independent risk factors. The model was adjusted for age, sex, and clinicopathological data, which showed a *p*-value smaller than 0.3 in the univariate regression. For each model, we provided 95% confidence intervals and the corresponding *p*-value. A similar analysis was repeated for both subgroups of the UICC staging.

Results were considered statistically significant if *p* < 0.05. All statistical analyses were conducted using R 4.0.2 (R Foundation for Statistical Computing, Vienna, Austria). The achieved power was 99.9% based on the observed effect size (validation set), the sample size (validation set), and α error probability of 0.05, indicating an adequate sample size (calculated using G*Power 3.1 [30]).

## 3. Results

### 3.1. Clinicopathological Characteristics of the Validation Collective

The clinicopathological data are summarized in Table 1. We enrolled 128 patients in our analysis, of which 78 (61%) were male. For 41 (32%) cases, metastasis was diagnosed during follow-up. Median follow-up time was 5.8 years. The mean age was 69 years, with a standard deviation of 12 years. The tumor was graded as pT4 in 19 (15%) cases and as pT3 in 109 (85%) cases. The lymph node status was positive in 53 (41%) of all cases. For 76 (59%) patients, the tumor was located on the right side. During follow-up, 53 (41%) patients died, which was caused by the tumor disease in 21 cases. Adjuvant chemotherapy was present in 52% of all cases, and the prevalence did not differ between the risk groups (*p* = 0.431). An overview of the clinicopathological characteristics of the two risk groups of BIg-CoMet is shown in Table 1. The characteristics of the training dataset are presented in Table S1.

### 3.2. Performance of BIg-CoMet

BIg-CoMet assigned 76 patients (59%) to the low-risk group and 52 patients (41%) to the high-risk group in the validation collective (*n* = 128). In the high-risk group, 31 out of 52 (60%) developed distant metastasis. In the low-risk group, 10 of 76 (13%) patients developed distant metastasis. Accordingly, the specificity was 75.9% (95% CI: 66.9–84.9%), the sensitivity was 75.6% (95% CI: 62.5–88.8%), the positive predictive value was 59.6% (95% CI: 49.5–69.0%), the negative predictive value was 86.8% (95% CI: 79.2–92.0%), and the proportion of correctly classified patients (accuracy) was 75.8% (95% CI: 67.4–82.9%).

### 3.3. Prognostic Analysis of the Validation Collective

**Figure 3:**
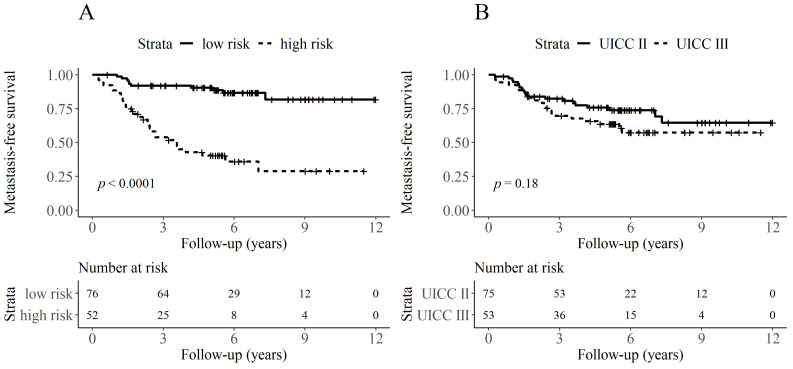
Kaplan-Meier curves for the occurrence of distant metastasis. Patients are stratified based on the classification of BIg-CoMet (*p* < 0.0001) (**A**) and the different UICC stages (*p* = 0.18) (**B**).

BIg-CoMet was a strong risk factor for the occurrence of distant metastasis, with a hazard ratio for the high-risk group of 6.9 (95% CI: 3.4–14.2, *p* < 0.001). In particular, in comparison to the UICC staging (Figure 3B), the Kaplan-Meier curves clearly illustrated a strong risk stratification effect (Figure 3A, log-rank-test: *p* < 0.001).

To adjust for other risk factors, we performed univariate Cox regression for age, sex, and clinicopathological characteristics (Table S2) and fitted a multivariable Cox model for all variables with a *p*-value smaller than 0.3 in univariate regression. Hence, for the multivariable model, the following variables were chosen: risk group, sex, age (continuous), tumor stadium, nodal status, lymphovascular invasion, tumor budding, location of tumor, and microsatellite (in)stability status. The risk group, defined by the BIg-CoMet classifier, showed a highly significant prognostic impact for metastasis-free survival with a hazard ratio of 5.4 (95% CI: (2.5–11.7), *p*-value < 0.001) for the high-risk group. Besides the BIg-CoMet classifier, the T-status and the stroma proportion (defined by groups) were independent risk factors (Table 2). In the high-risk group, there was a higher prevalence of death (high: 56% vs. low: 32%, *p* = 0.011) and a higher fraction of tumor-caused death (high: 29% vs. low: 8%, *p* = 0.004).

### 3.4. Analysis for UICC Subgroups

For patients with UICC II, we observed a good quality of classification (area under the curve (AUC): 0.756, sensitivity: 55.0%, specificity: 70.9%), which was even outperformed in the UICC III stages (AUC: 0.927, sensitivity: 95.2%, specificity: 84.4%). In patients in the high-risk group, metastasis occurred in 80%, whereas metastasis was detected only in 4% of patients in the low-risk group. In the UICC III subgroup, adjuvant chemotherapy was present in 79% of all cases. Adjuvant therapy was received by 89% in the low-risk group and by 68% in the high-risk group (*p* = 0.117).

**Figure 4:**
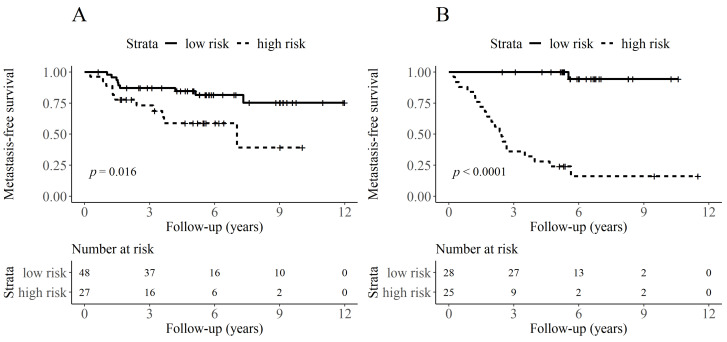
Kaplan-Meier curves for the occurrence of distant metastasis for both UICC stages. Patients are stratified based on the classification of BIg-CoMet for UICC II cases (*p* = 0.016) (**A**) and for UICC III cases (*p* < 0.0001) (**B**).

Separate Kaplan–Meier curves for metastasis-free survival for both groups underlined these results. Low- and high-risk groups, defined by BIg-CoMet, differed significantly with regard to the metastasis-free survival for both UICC grades (UICC II: log-rank-test *p* = 0.016, UICC III: log-rank-test *p* < 0.001) (Figure 4). The assignment of high-risk by BIg-CoMet was a significant prognostic predictor of the occurrence of distant metastasis in UICC II as well as UICC III cases (UICC II: HR = 2.9, 95% CI: 1.2–7.0, *p* = 0.021; UICC III: HR = 45.2, 95% CI: 6.0–340.8, *p* < 0.001).

## 4. Discussion

In this study, we demonstrated that BIg-CoMet, a CNN that used black and white histologic tumor images, outperformed the established histopathologic criteria (grading, t-status, nodal status, tumor budding, and the UICC staging) in predicting the occurrence of distant metastasis in locally advanced colon cancer. A multivariate Cox regression analysis showed the independence of the CNN predictions of clinicopathological characteristics and that the predictions were significantly associated with overall survival and colon cancer specific survival. The BIg-CoMet was validated on a different patient collective. The effect of risk stratification was consistently demonstrated in both UICC II and UICC III, although it was particularly pronounced in UICC III cases (Figure 4). We found small differences between the risk groups regarding adjuvant chemotherapy in UICC III. Although this difference did not reach significance, a certain influence of adjuvant therapy cannot be entirely ruled out. However, the difference presented in Figure 4B is considerably high. In our opinion, it seems unlikely that this effect is significantly influenced by an imbalance concerning the administration of adjuvant chemotherapy. Looking. e.g., at the results of the Levamisole and Fluorouracil trial [31], the probabilities of no recurrence events at 52 months were 66.1% and 50.8% in Lev + 5 FU and the control group. The metastasis-free survival rate in our study for high-risk cases was far worse and much better in the low-risk group (Figure 4B), indicating a true prognostic effect of BIg-CoMet stratification.

This exploratory finding has potential clinical implications. UICC II patients assigned to the high-risk group might benefit from adjuvant chemotherapy, while UICC III patients assigned to the high-risk group might benefit from adjusting the chemotherapy regime (e.g., a triple regime instead of a double regime: 5-Fluoruracil + Oxaliplatin + Irinotecan). [32,33]. Regardless of this, the very high negative predictive value in the UICC III cases is also of high interest. Only 3% of the assigned low-risk group developed distant metastasis. This information is very valuable for patients, and it can be used as an argument to avoid or adjust adjuvant chemotherapy if validated. In general, the benefit of chemotherapy is limited in the UICC stages II and III, and the benefit must be weighed against side effects [6,34,35,36]. The five-year disease-free survival rate is approximately 81% (without adjuvant chemotherapy) and 79% (with adjuvant chemotherapy) in UICC stage II and approximately 49% (without adjuvant chemotherapy) and 64% (with adjuvant chemotherapy) in UICC III [37]. Therefore, a more precise risk classification would be very desirable.

In addition to this study, a few studies have also examined prognostic forecasts on the basis of histological images. [38,39,40,41]. In all of these interesting studies, the prognostic value of the deep learning algorithm has been proven, although the methodology and study design differ considerably. In the first step, Kather et al. and Jiang et al. created small tiles of the H&E image and assigned them to different types of tissue using the CNN [39,40]. In the second step, prognostic analyses were calculated using these classifications. Similar to our study, Bychkov et al. [38], as well as Skrede et al. [41], performed the prognostic analyses by fitting a model directly on the image data, rather than first classifying tissue and computing tissue proportions. In this context, the performance (multivariate HR 5.4, 95% CI: 2.5–11.7 high-risk vs. low-risk group) of our established BIg-CoMet classifier is particularly impressive in consideration of the comparatively small size of the training set (*n* = 163). Skrede et al. trained on 828 patients (HR 3.04, 95% CI: 2.07–4.47, poor vs. good prognosis), and Bychkov et al. trained on 280 patients (HR 2.3, 95% CI: 1.79–3.03, high vs. low-risk patients) [38,41].

The result of the deep learning analyses is usually a black box regarding the question of which criterion or algorithmic signature led to the corresponding classifications. Besides the stratification of patients, a relevant strength of our study is that our model can make these predictions without consideration of histopathological features, such as the mitotic rate, the nuclear configuration of the tumor cells (or stromal cells), the configuration of individual tumor cells, or tumor-infiltrating lymphocytes, as they are not recognizable in the picture. Thus, it can be concluded that a binary image from the invasion front of the tumor contains enough structural information to classify patients. Features such as the tumor architecture, tumor stroma proportion, and tumor budding could be recognized. An association between the results of BIg-CoMet and the proportion of tumor stroma (groups) can be made. Both parameters were classified as independent predictors in the multivariate Cox regression model. The question arises as to whether BIg-CoMet made the risk classification based on previously unknown characteristics of the tumor architecture. If more in-depth studies can demonstrate this and identify the characteristics, these results could be re-translated into histological evaluation and have an impact on the follow-up of a patient and can hence support the therapy decision.

In comparison to this study, all previous studies used H&E images as input data. The preparation of the black and white images could be a source of interobserver variability, but we assume that they might be better suited for reproducible deep learning analyses between different centers by avoiding the considerable differences in H&E staining results of different laboratories [42]. Up to now, no standardized staining platform for H&E has received wide acceptance, and in most cases color pre-processing does not improve classification accuracy [43].

The study results presented here are consistent and very promising in identifying low-risk vs. high-risk patients, but it must be considered that this is a single center study. The most significant aspect in this regard is that all cases were processed by the Institute of Pathology and Molecular Diagnostics. For this reason, we cannot confirm that the methodology is independent of the institution.

Nevertheless, the results are also fascinating from another point of view. These results might be the first step in implementing deep learning into a modular and standardized principle. Instead of developing a comprehensive deep learning algorithm, a superior strategy could be to develop several specific algorithms for different images. Each model could be used for the image that has the highest informative value in a certain respect. For example, immunohistochemical staining for CD3 and CD8 could be the most suitable with regard to the immune profile. The immunoscore demonstrates the prognostic importance of the immune profile for the occurrence of metastasis [44,45]. The different (sub-)models can eventually be summarized in a comprehensive model. Depending on scientific advances, submodels can be swapped and adapted, making this a relatively flexible approach.

In consideration of the promising results presented here, the validation of different cohorts, as well as the investigation of its predictive value in a prospective setting, appears to be the next logical step on the way to clinical implementation.

## 5. Conclusions

Our results indicate that BIg-CoMet, a CNN that uses binary histologic tumor images, outperforms established histopathologic criteria in predicting the occurrence of distant metastasis in locally advanced colon cancer. Interestingly, the model was developed on images that primarily reflected the architecture of the tumor. Now, these promising data must be further validated and prospectively confirmed to implement BIg-CoMet in clinical routines.

## Figures and Tables

**Table 1:** Patient characteristics (validation set) and different BIg-CoMet risk groups.

Variable	Validation Set (*n* = 128)	BIg-CoMet Low Risk (*n* = 76)	BIg-CoMet High Risk (*n* = 52)	*p*-Value
Age, mean (SD), y	69 (12)	69 (12)	69 (12)	0.753
Sex, *n* (%)	-	-	-	0.764
Female	50 (39)	31 (41)	19 (37)	-
Male	78 (61)	45 (59)	33 (64)	-
Follow-up duration, median, years	5.8	5.9	5.5	0.780
Clinicopathological characteristics	-	-	-	-
Tumor stadium, *n* (%)	-	-	-	0.056
pT3	109 (85)	69 (91)	40 (77)	-
pT4	19 (15)	7 (9)	12 (23)	-
Nodal status, *n* (%)	-	-	-	0.278
Negative	75 (59)	48 (63)	27 (52)	-
Positive	53 (41)	28 (37)	25 (48)	-
Mean lymph node harvest (*n*)	21 (11)	20 (9)	21 (12)	0.911
Positive lymph nodes (*n*)	1.2 (2.3)	0.9 (1.7)	1.6 (2.9)	0.110
UICC, *n* (%)	-	-	-	0.278
II	75 (59)	48 (63)	27 (52)	-
III	53 (41)	28 (37)	25 (48)	-
Grading, *n* (%)	-	-	-	0.819
Low grade	76 (59)	44 (58)	32 (62)	-
High grade	52 (41)	32 (42)	20 (38)	-
Vascular invasion, *n* (%)	-	-	-	1.000
Negative	114 (89)	68 (89)	46 (88)	-
Positive	14 (11)	8 (11)	6 (12)	-
Lymphovascular invasion, *n* (%)	-	-	-	1.000
Negative	104 (81)	62 (82)	42 (81)	-
Positive	24 (19)	14 (18)	10 (19)	-
Tumor budding, *n* (%)	-	-	-	0.328
Bd 1	103 (80)	64 (84)	39 (75)	-
Bd 2	15 (12)	8 (11)	7 (13)	-
Bd 3	10 (8)	4 (5)	6 (12)	-
Location of tumor, *n* (%)	-	-	-	0.614
Right	76 (59)	47 (62)	29 (56)	-
Left	52 (41)	29 (38)	23 (44)	-
Microsatellite status, *n* (%)	-	-	-	0.896
Stable	115 (90)	69 (91)	46 (88)	-
Instable	13 (10)	7 (9)	6 (12)	-
Died, *n* (%)	-	-	-	0.011
Yes	53 (41)	24 (32)	29 (56)	-
No	75 (59)	52 (68)	23 (44)	-
Died of tumor, *n* (%)	-	-	-	0.004
Yes	21 (16)	6 (8)	15 (29)	-
No	107 (84)	70 (92)	37 (71)	-
Distant Metastasis, *n* (%)	-	-	-	<0.001
Yes	41 (32)	10 (13)	31 (60)	-
No	87 (68)	66 (87)	21 (40)	-
Tumor proportion, mean (SD)	0.358 (0.184)	0.376 (0.171)	0.326 (0.199)	0.143
Tumor proportion, *n* (%)	-	-	-	0.002
Low	21 (16)	6 (8)	15 (29)	-
Medium	80 (63)	56 (74)	24 (46)	-
High	27 (21)	14 (18)	13 (25)	-
Adjuvant Chemotherapy, *n* (%)	-	-	-	0.431
Yes	66 (52)	37 (49)	29 (56)	-
No	62 (48)	39 (51)	23 (44)	-

Abbreviations: BIg-CoMet = Binary ImaGe Colon Metastasis classifier, SD = Standard Deviation, UICC = Union Internationale Contre le Cancer.

**Table 2:** Multivariable Cox regression for occurrence of metastasis.

Variable	HR (95% CI)	*p*-Value
Sociodemographic characteristics	-	-
Age (continuous)	1.01 (0.98–1.04)	0.592
Sex (ref.: female)	1.2 (0.6–2.6)	0.626
Clinicopathological characteristics	-	-
Big-CoMet risk group (ref.: low)	5.4 (2.5–11.7)	<0.001
Tumor proportion (ref.: High)	-	-
Medium	0.4 (0.2–0.99)	0.047
Low	0.7 (0.3–1.7)	0.410
Tumor stadium (ref.: pT3)	2.6 (1.1–6.0)	0.029
Nodal status (ref.: negative)	0.9 (0.5–1.8)	0.838
Lymphovascular invasion (ref.: negative)	1.3 (0.6–3.2)	0.517
Tumor budding	1.6 (0.96–2.7)	0.069
Location of tumor (ref: right side)	1.5 (0.7–3.2)	0.245
Microsatellite status (ref.: Stable)	0.2 (0.02–1.2)	0.076

## Data Availability

The data presented in this study are available on request from the corresponding author.

## Supplementary Materials

The following are available online at https://www.mdpi.com/article/10.3390/cancers13092074/s1, Figure S1: In this figure the impact of the threshold on output of tumor and stroma in the conversion to a binary image is illustrated, Table S1: Patient characteristics (training set), Table S2: Simple Cox regression.
