# Supplementary material for: Deep Learning Prediction of Metastasis in Locally Advanced Colon Cancer Using Binary Histologic Tumor Images
Source: Cancers (Basel). 2021 Apr 25;13(9):2074. doi: 10.3390/cancers13092074 (PMC8123276; doi:10.3390/cancers13092074)
Supplement: Supplementary file 1 [file cancers-13-02074-s001.zip › cancers-1157366-supplementary.pdf]

Supplementary Materials: Deep Learning Prediction of Metastasis in Locally Advanced Colon Cancer Using Binary Histologic Tumor Images

Stefan Schiele, Tim Tobias Arndt, Benedikt Martin, Silvia Miller, Svenja Bauer, Bettina Monika Banner, Eva-Maria Brendel, Gerhard Schenkirsch, Matthias Anthuber, Ralf Huss, Bruno Märkl and Gernot Müller

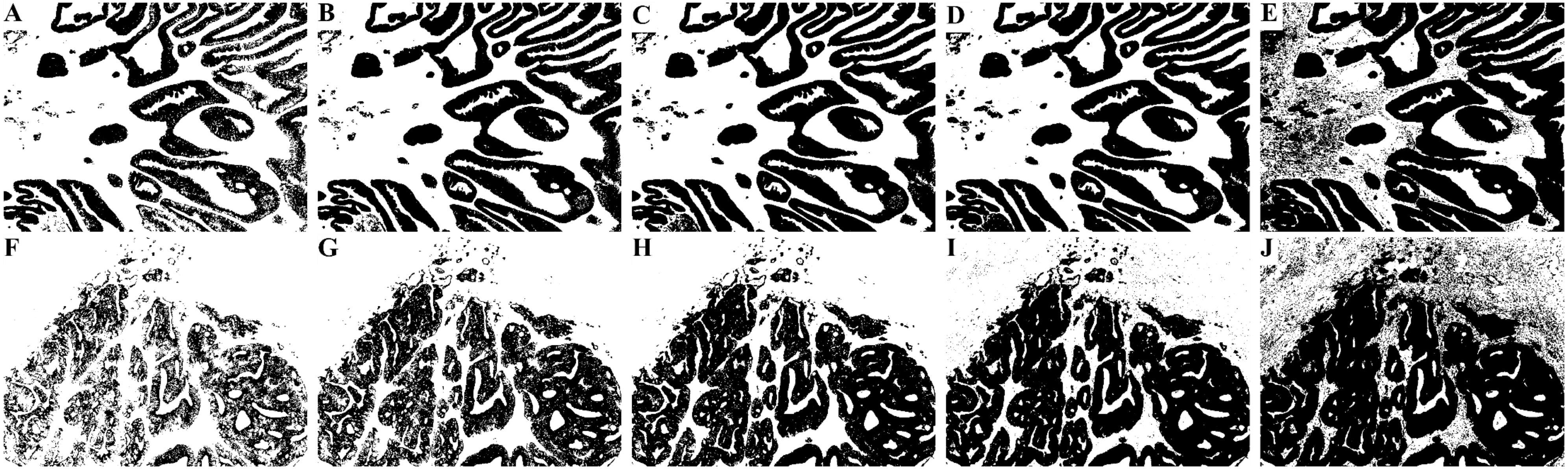

Figure S1. In this figure the impact of the threshold on output of tumor and stroma in the conversion to a binary image is illustrated. The images in the middle (**C**, **H**) use the automatically determined threshold of the IsoData algorithm. With each step to the left or right the threshold is shifted by 25 (**A**,**F** by −50; **B**, **G** by −25; **D**, **I** by +25; **E**, **J** by +50). Note, that while the changes in the threshold only have minimal impact on the translation of the tumor architecture, deviating from the automatic threshold introduces noise. With lower thresholds parts of the tumor are not recognized, whereas for higher thresholds stroma gets incorrectly classified as tumor.

Table S1. Patient characteristics (training set).

**Variable**	**Trainings Set (*n* = 163)** **Characteristics of the Population**
Age, mean (SD), y	69 (11)
Sex, *n* (%)	-
Female	68 (42)
Male	95 (58)
Follow-Up duration, median, years	5.2
**Clinicopathological characteristics**	
Tumor stadium, *n* (%)	-
pT3	113 (69)
pT4	50 (31)
Nodal status, *n* (%)	-
Negative	85 (52)
Positive	78 (48)
Mean lymph node harvest (*n*)	43.0 (20.0)
Positive lymph nodes (*n*)	2.0 (3.5)
UICC, *n* (%)	-
II	85 (52)
III	78 (48)
Grading, *n* (%)	-
Low grade	138 (85)
High grade	25 (15)
Vascular invasion, *n* (%)	-
Negative	140 (86)
Positive	23 (14)
Lymphovascular invasion, *n* (%)	-
Negative	122 (75)
Positive	41 (25)
Tumor budding, *n* (%)	-
Bd 1	104 (64)
Bd 2	36 (22)
Bd 3	23 (14)
Location of tumor, *n* (%)	-
Right	91 (56)
Left	72 (44)
Microsatellite status, *n* (%)	-
Stable	137 (85)
Instable	24 (15)
Died, *n* (%)	-
Yes	43 (26)
No	120 (74)
Died of tumor, *n* (%)	-
Yes	18 (11)
No	145 (89)
Distant Metastasis, *n* (%)	-
Yes	64 (39)
No	99 (61)
Tumor proportion, mean (SD)	0.507 (0.111)
Tumor proportion, *n* (%)	-

Low	0 (0)
Medium	105 (65)
High	57 (35)
Adjuvant Chemotherapy, *n* (%)	-
Yes	69 (42)
No	94 (58)
Abbreviations: SD = Standard Deviation, UICC = Union Internationale Contre le Cancer	

Table S2. Simple Cox regression.

**Sociodemographic Characteristics**	***p*-value**
Age (continuous)	0.039
Sex	0.287
Clinicopathological Characteristics	-
Risk group	<0.001
Tumor stadium	0.001
Nodal status	0.183
Mean lymph node harvest (*n*)	0.373
Grading	0.722
Vascular invasion	0.315
Lymphovascular invasion	0.059
Tumor budding	0.014
Location of tumor	0.079
Microsatellite status	0.094
Tumor proportion	0.003
Adjuvant Chemotherapy	0.750
